# The Use of Flexible Ultrasound Transducers for the Detection of Laser-Induced Guided Waves on Curved Surfaces at Elevated Temperatures

**DOI:** 10.3390/s17061285

**Published:** 2017-06-04

**Authors:** Tai Chieh Wu, Makiko Kobayashi, Masayuki Tanabe, Che Hua Yang

**Affiliations:** 1 College of Mechanical and Electrical Engineering, National Taipei University of Technology, Taipei 10608, Taiwan; chyang@ntut.edu.tw; 2 Faculty of Advanced Science and Technology, Kumamoto University, Kumamoto 8608555, Japan; kobayashi@cs.kumamoto-u.ac.jp (M.K.); mtanabe@cs.kumamoto-u.ac.jp (M.T.)

**Keywords:** flexible ultrasonic transducer, laser ultrasonic technique, laser ultrasonic visualization, material characterization, defect detection, Non-destructive testing, high temperature measurement

## Abstract

In this study, a flexible ultrasonic transducer (FUT) was applied in a laser ultrasonic technique (LUT) for non-destructive characterization of metallic pipes at high temperatures of up to 176 °C. Compared with normal ultrasound transducers, a FUT is a piezoelectric film made of a PZT/PZT sol-gel composite which has advantages due to its high sensitivity, curved surface adaptability and high temperature durability. By operating a pulsed laser in B-scan mode along with the integration of FUT and LUT, a multi-mode dispersion spectrum of a stainless steel pipe at high temperature can be measured. In addition, dynamic wave propagation behaviors are experimentally visualized with two dimensional scanning. The images directly interpret the reflections from the interior defects and also can locate their positions. This hybrid technique shows great potential for non-destructive evaluation of structures with complex geometry, especially in high temperature environments.

## 1. Introduction

Structures with curved surfaces such as pipelines or pressured vessels that are required to operate at elevated temperatures are commonly seen. Defects due to corrosion, erosion, and cracks may lead to catastrophic outcomes. Nondestructive testing (NDT) techniques have been continuously developing for early detection of possible defects to ensure a structure’s integrity. Among them, ultrasound techniques are widely used because of their advantages in terms of cost effectiveness, being free of radiation, and their versatility to be applied under numerous different conditions. In recent years, ultrasonic techniques (UTs) have been based on guided wave detection and were developed for the detection of subsurface and interior faults. However, UTs have some limitations such as their range of working temperatures and incomplete surface conductivity. Currently, when using ultrasonic wave detection at high temperatures, optical interferometers have overcome the temperature shortcoming, but their sensitivity is low and the preparation of a smooth specimen surface is necessary. Therefore, a more robust inspection technique, which can be applied to more complex geometry at high temperatures is very desirable.

A thorough review of various piezoelectric materials and bonding techniques was compiled by Kažys [1]. In the past decade, many researchers have developed high temperature sensors for inspection and condition monitoring [2,3]. The most often used piezoelectric materials in manufacturing ultrasonic transducers and their applications are as follows: first, lithium niobate (LiNbO_3_) single crystals are one of the most well-known elements due to its high Curie temperature threshold of T_c_ 1142–1210 °C and its ideal piezoelectric element performance. It has been used in high temperature transducers for ultrasonic testing at 400 °C since 1989 [4]. Since then, a series of studies have focused on the improvement of its working temperature by using the dice and fill method [5,6,7,8]. However, LiNbO_3_ has low thermal shock durability due to its single crystal structure. In addition, LiNbO_3_ cannot sustain high temperatures for long terms because of oxidation losses. Bismuth titanate (BIT) and modified bismuth titanate (MBIT) are also commonly used as the piezoelectric element for direct contact ultrasonic transducers. These materials exhibit a low dielectric constant, low dielectric losses and their properties are stable up to very high temperatures. In earlier applications, BIT and MBIT successfully served as a tool for ultrasonic thickness monitoring [9] and pipeline defect inspection [10] at temperatures of up to 350 °C. The most popular piezoelectric material used for manufacture of ultrasonic transducers is lead zirconate titanate (PZT). It has very good electromechanical properties, but is compromised by having a relatively low T_c_ threshold/tolerance of 350 °C which is far below that of most other piezoelectric materials such as LiNbO_3_, BIT, GaPO_4_, etc. An NDT device fabricated with a combination of PZT and LiNbO_3_ or PbTiO_3_ (PT) and PZT as a composite piezoelectric material was successfully tested by Kobayashi et al. for uses at elevated temperatures [11,12]. In their studies, a sol-gel composite was developed to solve the problems caused by high temperatures [13]. A sol-gel composite consists of a piezoelectric powder phase and a high dielectric constant sol-gel phase. It can be applied to the piezoelectric material [14] and thick film ultrasonic transducer by using the sol-gel spray technique [15,16,17]. With this technique, a piezoelectric ceramic film can be easily fabricated at the desired location on the surface of molds or dies through a shadow mask. In addition, the film can be sprayed onto a thin plate substrate to be used as a flexible ultrasonic transducer (FUT) which takes advantages of its curved surface adaptability, high temperature durability, broad band frequency response and high signal-to-noise ratio [18,19,20,21]. In this process, a FUT with PZT/PZT composite film is applied to receive the laser induced guided waves propagating on the curved surface at high temperature. The acoustic performance of this sensor has been verified in previous studies [22,23,24].

A laser-induced ultrasonic wave is one of the most powerful techniques in the field of nondestructive evaluation (NDE) or structure health monitoring (SHM). The ability of contact-free excitation, multi-mode guided wave generation, and rapid inspection of various structures are among the main advantages of laser ultrasound [25,26,27,28]. In fact, when a pulsed laser beam is irradiated onto a solid, those waves can be considered a versatile means for the evaluation of the elastic properties of materials. In our previous studies, laser-induced ultrasound was used to measure the dispersions of guided waves and characterize material properties such as the material’s hydrogen concentration [29], solid oxide full cells [30] and nickel aluminum coatings [31]. 

Laser ultrasound imaging (LUI) is a cutting-edge inspection technique, which employs a pulsed laser to scan over the area of interest and visualize the resulting wave propagations. It effectively shortens the analysis time and satisfies the requirements for easy interpretation of ultrasonic propagation without reference data. In previous studies [32,33,34,35,36,37,38], a Q-switched pulsed laser and a galvano-motorized mirror were utilized to generate guided waves, and a PZT ultrasonic transducer used as a receiver was fixed on to the specimen during scanning. The wave field visualization application included measurement of the phase and group velocities of Lamb waves, wave propagation on different structures and defect detection, and all these parameters had been successfully demonstrated. When analyzing defect detection by using LUI, the research focused on the interaction of the laser-generated Rayleigh wave on surface-breaking cracks [39,40]. However, any inner defect of a piece of equipment should be taken seriously during any industrial inspection.

The main objective of this work is material characterization and defect detection based on laser-induced ultrasound on a metallic pipe at high temperature. The dispersion curves can represent the material characterization with the change of temperature by using laser ultrasonic technique (LUT). Additionally, defect detection can be performed by using LUI to monitor the dynamic wave propagation behaviors. A PZT/PZT based FUT is applied to be a sensor due to its self-alignment to the curved surface and high temperature durability. The outline of this research is as follows: Section 2 briefly describes the fabrication process of the FUT and its ultrasonic performance testing. The experimental setup and the sketches for the LUT and LUI methods are shown in Section 3. Section 4 illustrates the results from the LUT and LUI experiments. Section 5 presents our/the conclusions of this work.

## 2. Flexible Ultrasound Transducer

**Figure 1:**
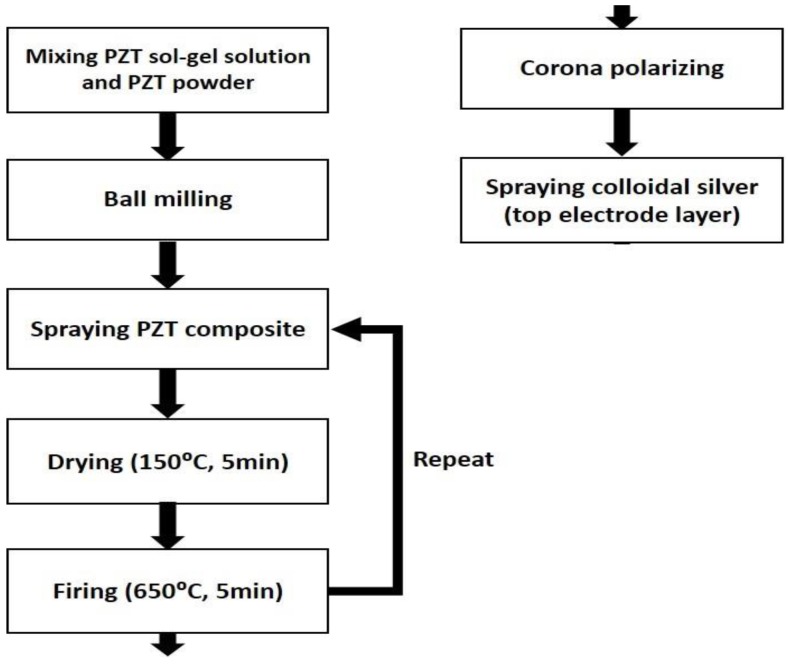
FUT fabrication process.

The PZT/PZT transducer is fabricated by utilizing the sol-gel spray method which is outlined in Figure 1. In this process, a submicron fine PZT powder is first dispersed into the sol-gel solution by ball milling. The liquid mixture is sprayed directly onto a 40 mm × 40 mm, 50 μm-thick piece of stainless steel substrate (SS304) by an airbrush to form a layer of coating. The coated layer on the substrate is then dried by a plate heater at 150 °C for 5 min and then baked in a furnace at 650 °C for another 5 min. The coating and thermal processes are repeated until the sprayed film reaches the desired thickness. Later on, the coated film is electrically poled by using a corona discharging technique. For corona poling, positive high voltage power is fed into a sharp, thin needle that is above the film. After polarization through the corona discharging at room temperature, a colloidal silver is sprayed on to the sensor area of the PZT/PZT that was layered by the air brush.

**Figure 2:**
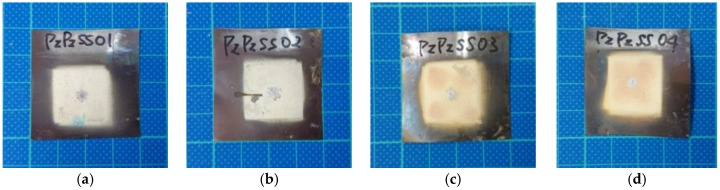
Fabricated FUTs: (**a**) PzPzss01; (**b**) PzPzss02; (**c**) PzPzss03; (**d**) PzPzss04.

Figure 2 shows four fabricated FUTs labeled as PzPzss01, PzPzss02, PzPzss03 and PzPzss04 corresponding to sprayed film thicknesses of 80, 72, 146 and 138 μm, respectively. 

**Figure 3:**
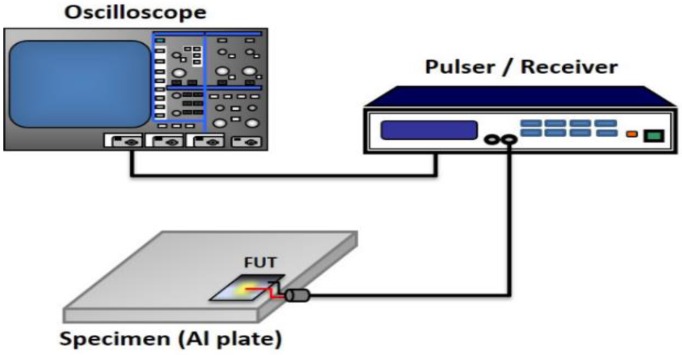
Schematic diagram of ultrasonic performance for FUT.

The performance of the FUTs was further tested after the fabrication process using the experimental setup as shown in Figure 3. A pulser/receiver (Panametric 5900PR, Olympus, Waltham, MA, USA) is used to drive the FUTs in the pulse/echo mode. The testing specimen is an aluminum plate with a thickness of 8.5 mm. The detected signal was recorded with a digital oscilloscope (WR44Xi, LeCroy, Thief River Falls, MN, USA) and transferred to a computer for data processing. 

**Figure 4:**
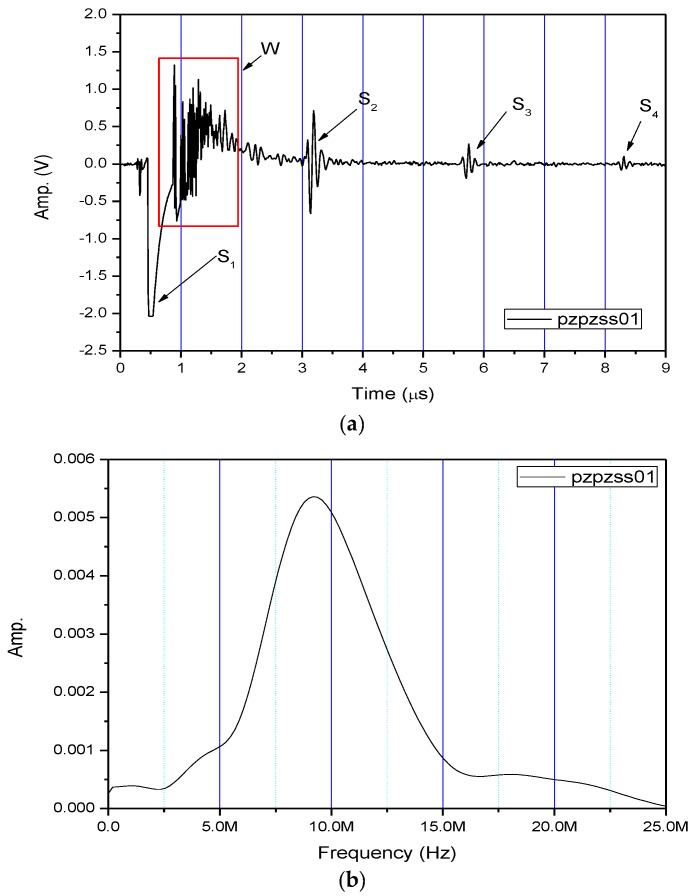
Performance of Pzpzss01: (**a**) Time domain signal; (**b**) spectrum.

**Figure 5:**
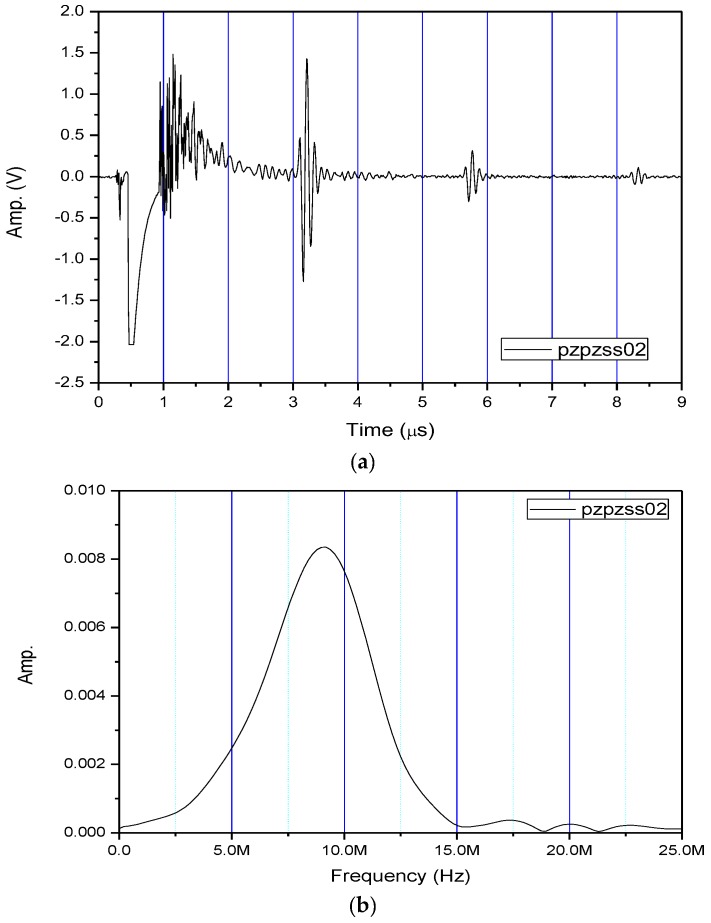
Performance of Pzpzss02: (**a**) Time domain signal; (**b**) spectrum.

**Figure 6:**
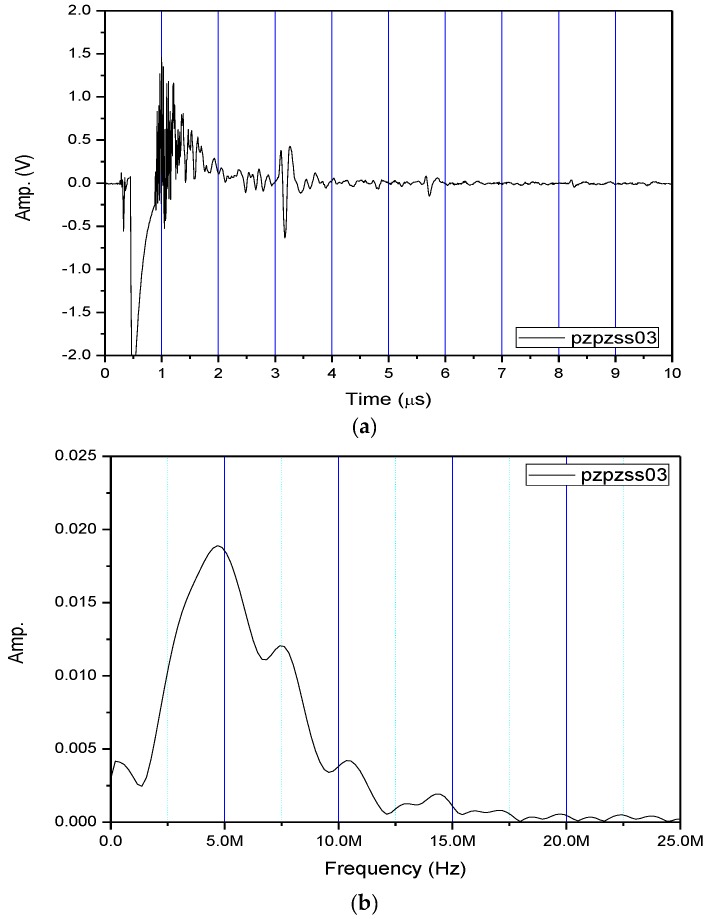
Performance of Pzpzss03: (**a**) Time domain signal; (**b**) spectrum.

**Figure 7:**
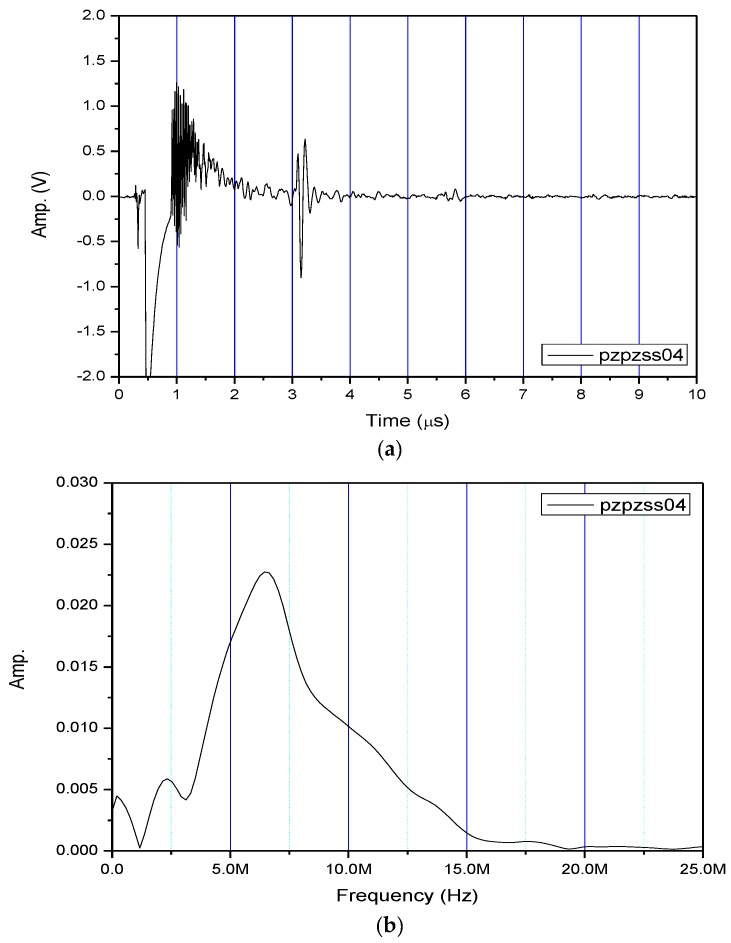
Performance of Pzpzss04: (**a**) Time domain signal; (**b**) spectrum.

Figure 4a shows the received pulse/echo signal by using the Pzpzss01 FUT transducer with the experimental setup shown in Figure 3. In this time domain trace, S1 corresponds to the initial pulse, S2, S3, and S4 for the first, second and third reflections from the bottom surface of the plate. The signals in the window are designated as W which are multiple reflections between the top and bottom surfaces of the FUT substrate. Figure 4b shows the frequency spectrum after a Fast Fourier Transform (FFT) from the S2 signal. With a film thicknesses of 80 μm on the Pzpzss01 sensor, the central frequency is 9.18 MHz. Similar signals with good SNR for the other 3 FUTs are shown in Figure 5, Figure 6 and Figure 7 with central frequencies of 9.18 MHz, 4.89 MHz and 6.45 MHz for the Pzpzss02, Pzpzss03 and Pzpzss04, respectively. As shown in Table 1, the central frequency of the FUT decreases as the sprayed film thickness increases. With this information, we can customize the central frequency by controlling the film thickness of the FUT. 

## 3. Laser Ultrasonic Technique (LUT) Tests

**Figure 8:**
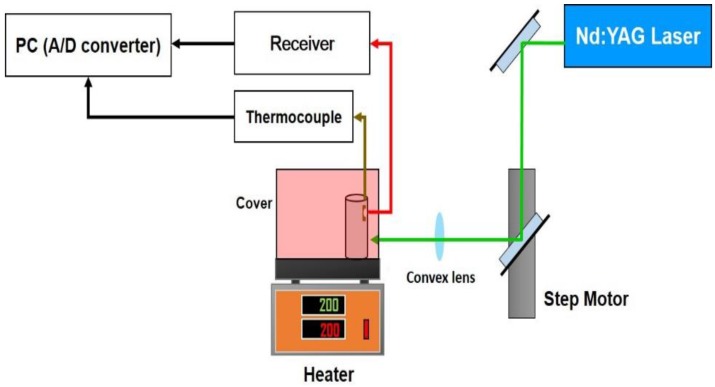
Experimental setup for the LUT with FUT.

In the LUT test, dispersion spectra of guided acoustic waves traveling along a metal tube at elevated temperatures are measured by laser-generation and FUT detection. A stainless steel pipe with an outer diameter of 48 mm and a thickness of 2.2 mm is tested with the LUT while Pzpzss02 is used as an ultrasound detector. Figure 8 is a schematic for the experimental configuration of the LUT system at elevated temperatures. A pulsed Nd:YAG laser (Quantel, Brilliant B, Les Ulis, France) with a wavelength of 532 nm, a duration time of 6.6 ns, and an energy output of about 100 mJ is used for the ultrasound generation. The sensor is attached with a small amount of ultrasonic coupler on the surface of the pipe and fixed using a high temperature durable tape made of polytetrafluoroethylene (PTFE). Meanwhile, a thermocouple is also attached on to the interior of the pipe for monitoring the temperature. The stainless steel pipe is heated with a hot plate and covered with an asbestos cover to maintain temperatures of 25 °C, 65 °C, 92 °C, 119 °C, 148 °C and 176 °C. When the temperature reaches a steady state, the scanning stage controlled by a computer drives a mirror to scan a Nd:YAG laser beam along the axial direction of the pipe. A computer with a fast analog to digital converter (ADC) is used for controlling the scanning stage, waveform acquisition, temperature recording and further signal processing. Guided waves are generated with the pulsed laser to propagate throughout the heated stainless steel pipe.

**Figure 9:**
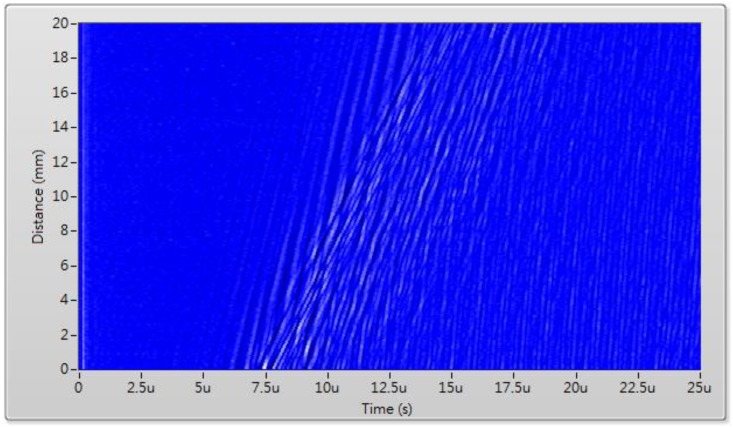
B-scan data for the stainless steel pipe with the LUT/FUT at room temperature.

By collecting the waveforms at each step, Figure 9 shows the set of B-scan data collected at room temperature with a total scanning distance of 20 mm with 200 steps. The B-scan data is further processed with a two-dimensional fast Fourier transform (2D-FFT) signal processing. During the 2D-FFT, the first FFT is taken with respect to time, and the second FFT with respect to the scanning position. The 2D-FFT transforms the B-scan data into ultrasound amplitude as a function of frequency (*f*) and wavenumber (*k*). A peak-detection routine is used to find the trajectories of peak amplitudes in the f-k space. Finally, dispersion curves in the form of ultrasound phase velocity (*V*) versus frequency are obtained with the aid of the relation *V = 2πf/k*. 

## 4. Laser Ultrasonic Imaging (LUI) Tests

**Figure 10:**
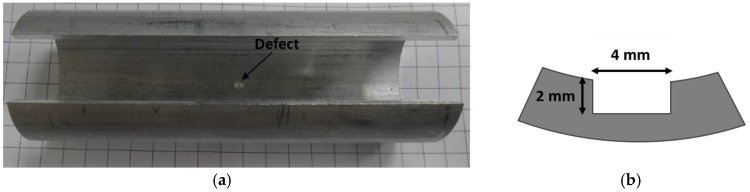
(**a**) Aluminum pipe with defect; and (**b**) schematic graph of defect.

**Figure 11:**
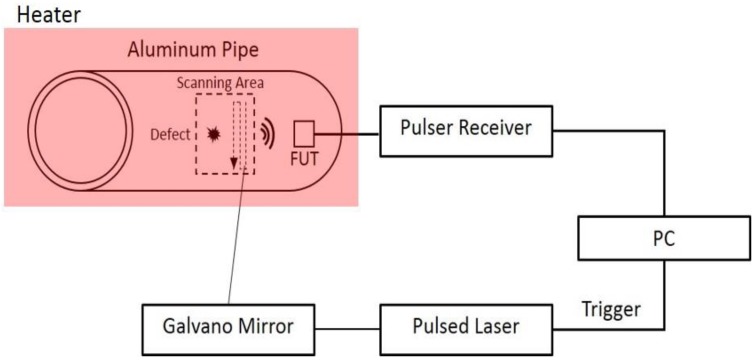
Schematic experiment setup graph of LUI with FUT.

**Figure 12:**
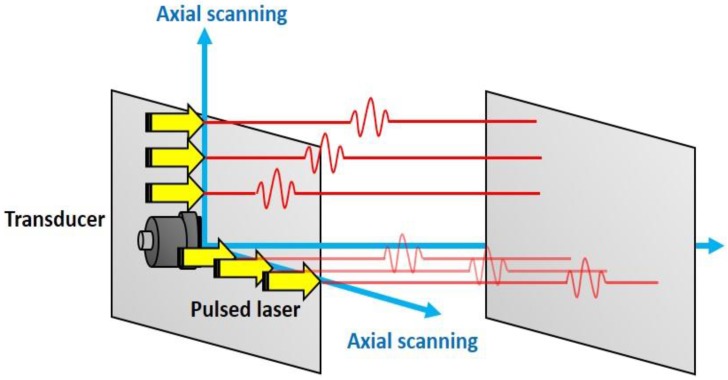
LUI imaging processing.

An aluminum pipe with an outer diameter of 50 mm and a thickness of 3 mm, which has an interior crack, is used as a specimen for LUI as shown in Figure 10. Figure 11 illustrates the experimental setup. A pulsed Nd:YLF laser (Optowave, Awave, Ronkonkoma, NY, USA) with a wavelength of 1064 nm, a maximum repetition rate of up to 20 kHz, a pulse energy of about 2 mJ, with a 0.7 mm beam diameter is employed to generate guided waves. The Pzpzss02 sensor is attached on to the surface of pipe with a small amount of ultrasonic coupler and fixed using PTFE tape. The scanning mechanism is a two axis galvano-mirror, which is controlled by the computer to scan the Nd:YLF laser beam onto the exterior surface of the pipe. The specimen is heated with an asbestos cover and operated at room temperature, 75 °C and 95 °C. A computer with a fast analog to digital converter is used for signal acquisition, scanning control and image post-processing. With the LUI system, the pulsed laser scans over the area of interest and the detected signals pile up into a data cube with the dimensions of (*x*, *y*, *t*) as shown in Figure 12. The data cube is time-gated at various elapsed times, so a series of pictures are created. With the aid of reciprocal theorem, these pictures represent many instantaneous frames representing wavefronts generated by the FUT that were detected at the scanning area. The measured points are arranged in a 470 × 150 grid with a pitch of 0.1 mm at room temperature, and a 150 × 100 grid with a pitch of 0.1 mm at 75 °C and 95 °C. An imaging process to superimpose all the frames is employed in order to specify the position of defect.

## 5. Results and Discussion

### 5.1. LUT Testing Results

**Figure 13:**
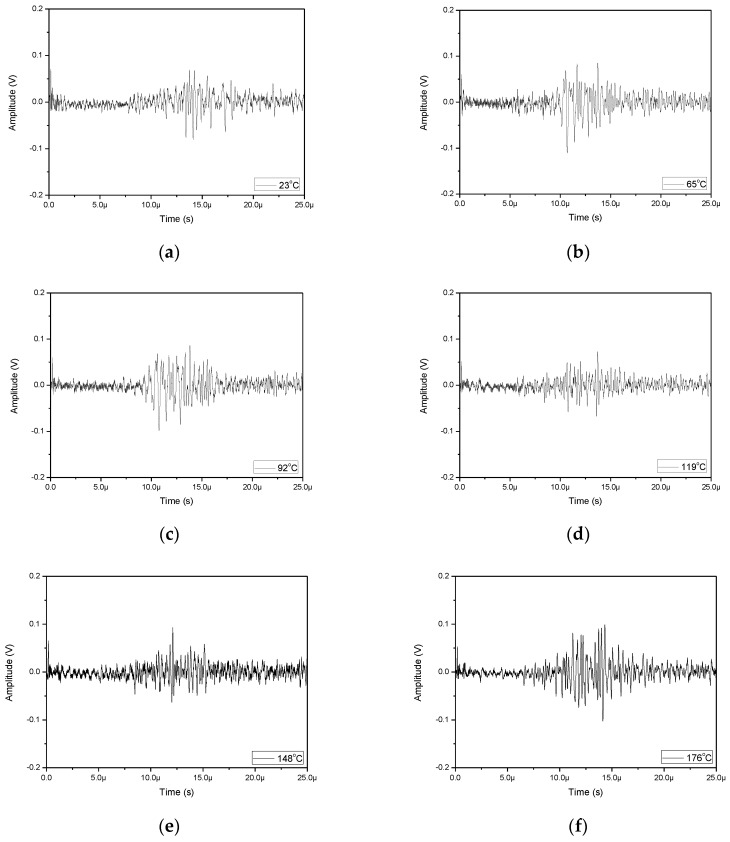
Measured signals in LUT/FUT at (**a**) 25 °C; (**b**) 65 °C; (**c**) 92 °C; (**d**) 119 °C; (**e**) 148 °C; and (**f**) 176 °C.

**Figure 14:**
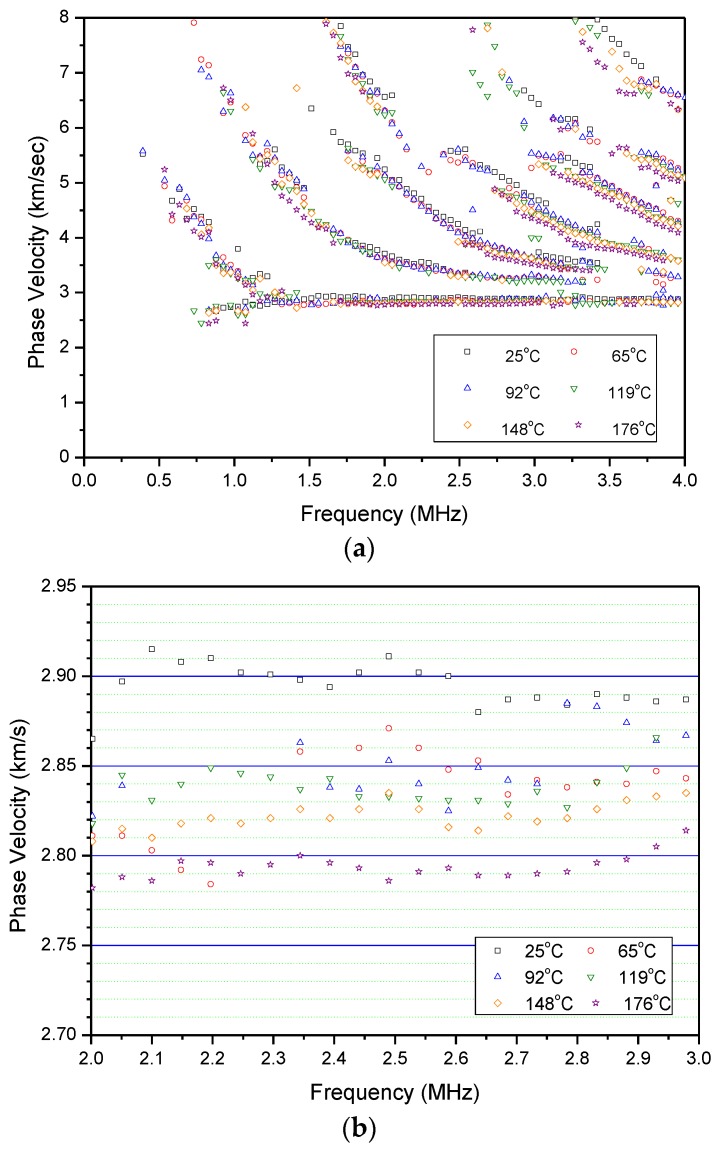
(**a**) Measured dispersions at various temperatures; and (**b**) a zoom-in for the measured dispersions.

Figure 13 shows waveforms generated with the pulsed Nd:YAG laser in the LUT and detected with the FUT for temperatures from 25 °C to 176 °C. With B-scan and 2D-FFT processing, Figure 14a shows the measured dispersion spectrum for the stainless steel pipe at various temperatures. Dispersion spectra with obvious multi-mode structures from the guided waves propagating through the sample at elevated temperatures are obtained. The dispersion curves shift in a downward trend towards the lower frequencies and lower phase velocities as the temperature increases. With the aid of a zoomed-in graph of the data shown in Figure 14b, the phase velocity of surface wave is noticeably reduced as the temperature increases. The measured surface wave velocity is 2900 m/s at 25 °C and 2780 m/s at 176 °C, corresponding to a reduction of 100 m/s due to the increased temperature. Compared with other ultrasound probes, a FUT mounted on a pipe for can last as long as 3 h which shows that this system has the capability to continuously inspect, monitor and gather data in an elevated temperature environment.

### 5.2. LUI Testing Results

**Figure 15:**
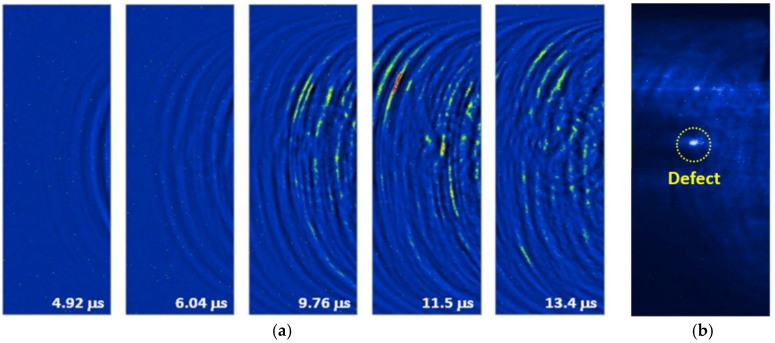
(**a**) Frames of guided waves propagating on the stainless steel pipe with an interior defect; and (**b**) the processed image by accumulating the intensity of each of the frames at room temperature.

Figure 15a shows visualized images for the laser-generated/FUT-detected guided wave propagation along the pipe at room temperature. Here, at least two guided wave modes can be seen propagating along the pipe by observing different elapsed times. One is a faster mode (L(0,2)) and the other is a slower mode (L(0,1)) with a larger amplitude. The ultrasonic waves passed through a hole with a diameter of 4 mm, and are scattered in the image at 9.76 μs. The defects relative position is ensured though the dynamic imaging and our understanding of guided wave propagation behavior. Besides, the defect can be emphasized by further signal processing and can be displayed in a static image. We accumulated the amplitudes of each time domain signal at the same *x* and *y* position for every image. Figure 15b displays the processed static image obtained from the dynamic result with a total of 500 frames. By accumulating the energy of each frame, the defect region is able to be more easily seen because the reflected and scattered waves primarily originated from the defect itself. 

**Figure 16:**
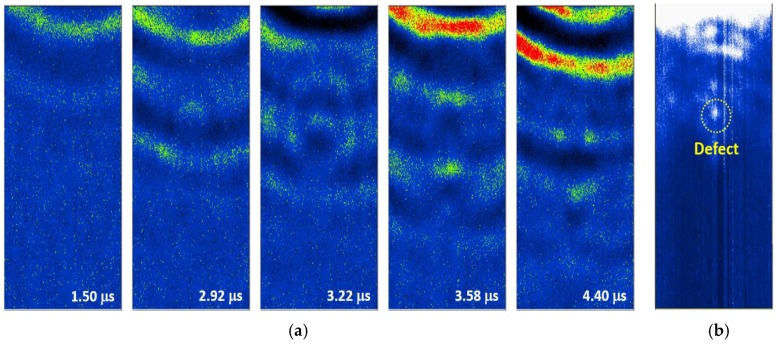
(**a**) Frames of guided waves propagating on the stainless steel pipe with an interior defect and (**b**) the processed image gathered by accumulating the intensity of each of the frames at 75 °C.

**Figure 17:**
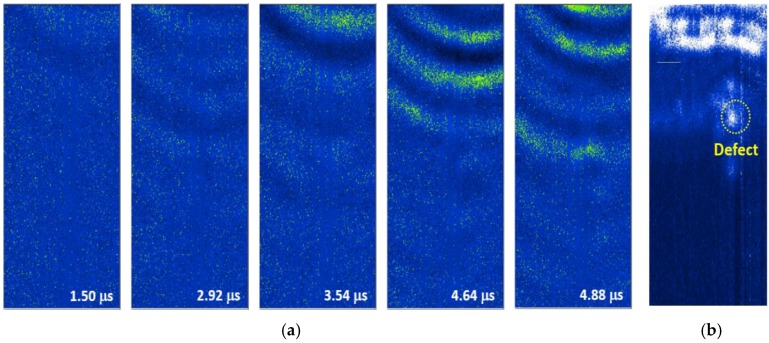
(**a**) Frames of guided waves propagating on the stainless steel pipe with an interior defect; and (**b**) the processed image gathered by accumulating the intensity of each of the frames at 95 °C.

Figure 16a and Figure 17a present the wave propagation images which were extracted from the LUI at 75 °C and 95 °C. With the relative small scanning area compared with the room temperature measurements, the two guided wave modes can be observed clearly at different elapsed times. For the 75 °C LUI test, the wave propagations of (L(0,2)) mode and (L(0,1)) mode are visualized at 1.50 μs and 3.58 μs, respectively. Both of them show the reflections and the changes in the wave fronts that resulted from the interior defect. In contrast with dynamic wave propagation behavior, the change in the waveform can be observed only when the slower mode (L(0,1)), passes through the defect at the 95 °C point of the LUI test. To enhance the defects location with respect to the scanning area, the post-processed images are shown in Figure 16b and Figure 17b. The defect comes out clearly by cumulating 500 frames of both processed static images. Some energy appeared on the top of the reconstructed image because the sensor was placed close to the edge of scanning area. There are also a few anomalies that can be seen in the reconstructed image because of the imperfect contact between the BNC cable and the FUT anode during the scanning process. 

## 6. Conclusions

This paper demonstrates that the integration of FUT’s and laser-induced ultrasound applied during material characterization and defect detection for curved surfaced structures at high temperatures has merit. With FUT fabrication, the frequency response can be controlled through the sol-gel spraying process. The PZT/PZT film thickness is inversely proportional to its central frequency. In this study, a 9.18 MHz FUT with a film thickness around 80 μm was utilized to be the ultrasonic receiver in two experiments. For material characterization, the multi-mode dispersion spectrum of a stainless steel pipe can be obtained through signal processing at high temperatures of up to 176 °C. The guided wave modes shifted at a downward trend towards the lower frequencies and lower phase velocities when the temperature was increased. Furthermore, the FUT was able to continuously measure at elevated temperatures for as long as 3 h. For defect detection, although the signals were affected by the thermal noise from the heater and an inadequate connection to the FUT, the dynamic wave propagation behaviors of an aluminum pipe with an interior defect were still visualized through two-dimensional scanning. Although the reflections from the interior defect become weaker when the temperature is raised, the defect can be still highlighted by compiling each frame obtained from LUI. The performance results of LUI with the FUT were outstanding and they included curved surface analysis feasibility as well as the ability to quickly scan specific areas and easy identification of any defects from the guided wave propagation images.

## Figures and Tables

**Table 1:** Film thickness and central frequency of the FUTs.

Label	Film Thickness (μm)	Central Frequency (MHz)
PzPzss01	83	9.18
PzPzss02	72	9.18
PzPzss03	146	4.89
PzPzss04	138	6.45
